# Sensitive marker for evaluation of hypertensive heart disease: extracellular volume and myocardial strain

**DOI:** 10.1186/s12872-020-01553-7

**Published:** 2020-06-15

**Authors:** Junqiao Niu, Mu Zeng, Yan Wang, Jun Liu, Hui Li, Shanshan Wang, Xiaoyue Zhou, Jia Wang, Yanyu Li, Feng Hou, Junwen Zhu

**Affiliations:** 1 Radiological imaging Center, People’s Hospital of Xinjiang Uygur Autonomous Region, Urumqi, 830001 China; 2 Department of Radiology, The Second Xiangya Hospital, Central South University, Changsha, Hunan China; 3 MR Collaboration, Siemens Healthineers Ltd, Shanghai, China; 4 Cardiopulmonary function department, Affiliated Cancer Hospital of Xinjiang Medical University, Urumqi, China

**Keywords:** HHD, CMR, ECV, Myocardial strain

## Abstract

**Background:**

Evaluation of tissue fibrosis and myocardial hypertrophy in left ventricular (LV) remodeling is the basis of post-treatment evaluation of hypertensive heart disease (HHD). Extracellular volume (ECV) and myocardial strain parameters can indirectly reflect the changes of both. Our objective was to analyze the characteristics of ECV and strain parameters in LV myocardium of HHD with varying degrees of systolic dysfunction, and to explore the changes of both after treatment for hypertension.

**Methods:**

A total of 62 HHD patients were divided into 3 groups according to ejection fraction (EF < 30, 30%≦EF < 50%, EF≧50%). Twenty-one of these patients underwent cardiac magnetic resonance (CMR) reexamination more than six months after receiving antihypertensive medication. The initial T1 time and post-enhancement T1 time of each segment were measured, and the ECV was calculated. Radial strain (RS), circumferential strain (CS) and longitudinal strain (LS) of LV were measured by cvi42 software, and the differences in CMR parameters between different groups and before and after treatment were compared.

**Results:**

①The mean, basal and middle ECV value of HHD groups with different EF were all higher than that of the control group (*P* < 0.05), but the difference between HHD groups was not statistically significant. ②With the decrease of EF, the absolute value of both the global or local strain decreased. Strain is related to LVMI and ECV. ③In general, ECV, global RS (GRS) and global CS (GCS) improved after treatment, but the improvement of LS impairment in HHD patients is difficult.

**Conclusions:**

ECV and myocardial strain parameters are more sensitive to myocardial abnormalities, and ECV, GRS and GCS are more sensitive to treatment. However it is difficult to improve longitudinal strain impairment in HHD patients. ECV and myocardial strain parameters can be used as good makers for long-term monitoring of the efficacy of HHD patients.

## Introduction

HHD is a structural and functional abnormality of the heart caused by a long-term increase in systemic circulating arterial pressure. Its pathophysiological basis is cardiac remodeling, which including the development of left ventricular hypertrophy (LVH) and diffuse interstitial fibrosis [1]. LVH is an independent risk factor for essential hypertension cardiovascular events [2–4]. Myocardial fibrosis is the most important predictor of diastolic dysfunction [5] and is also involved in the development of systolic heart failure in hypertensive patients [6]. Therefore, the evaluation of tissue fibrosis and cardiomyocyte hypertrophy in left ventricular remodeling is important for post-treatment evaluation of HHD. ECV and myocardial strain parameters can be obtained non-invasively using CMR imaging. In CMR studies, ECV can be used as an important indicator to identify myocardial diffuse fibrosis, early myocardial fibrosis or mild myocardial abnormalities. Several studies have suggested an increase in diffuse fibrosis in hypertensive patients as demonstrated via CMR measurements of ECV. However, these changes were small and only occurred in patients with LVH [7]. CMR myocardial strain measurements can be used to monitor the whole heart or local myocardial systolic dysfunction at early disease stages. Multi-parametric CMR methods should be ideal for monitoring these patients given critical relationship between strain, fibrosis, and cardiomyocyte hypertrophy.

Multiple studies have shown that hypertension medication can reverse LVH [8, 9], but the risk of heart failure remains after return of LVH [10, 11]. CMR measurements during animal model studies demonstrated changes in cardiomyocyte hypertrophy and interstitial fibrosis following treatment [12]. Additional clinical studies are critically needed to explore the relationship between myocardial structure and function by CMR, particularly following therapy in patients with HHD. The purpose of our study was to compare CMR myocardial ECV and strain parameters in HHD patients with different degrees of left ventricular dysfunction and also compare these myocardial ECV and strain parameters before and after antihypertensive drug therapy.

## Methods

### Patients

The subjects were patients with hypertension who underwent contrast-enhanced CMR scans in the out-patient and in-patient departments of Xiangya Second Hospital of Central South University between January 2016 and September 2019. At the same time, LVH is required in subjects. Hypertension was determined on the basis of blood pressure ≥ 140mmhg systolic or ≥ 90mmhg diastolic on three different days. The reference criterion for LVH was LVMI, a measurement parameter of cardiac magnetic resonance imaging, > 81 g/m^2^ in men,> 61 g/m^2^ in women [13]. Exclusion criteria for this study included cardiomyopathy, secondary hypertension, arrhythmia, coronary heart disease, significant valvular heart disease, a history of diabetes, a history of malignant tumors, a history of sleep apnea, and severe kidney disease when examinees were known to have other causes. Among the included patients, 21patients received CMR reexamination after regular use of antihypertensive drugs for more than 6 months. In this study, 26 healthy persons, age matched to the above HHD group, with normal blood pressure and no history of hypertension, served as a control and underwent identical CMR examination protocol. This study was approved by the ethics committee of Xiangya Second Hospital of Central South University, and all subjects signed an informed consent.

### CMR protocol

Imaging studies were performed on a 3 T scanner (MAGNETOM Skyra, Siemens Healthcare, Erlangen, Germany) with an 18-channel body coil combined with the spine coil. Scanning protocols included both the LV cine sequence using a segmented balanced steady-state free-precession (bSSFP) acquisition performed at the end of inspiration and the pre/post T1-mapping sequences using the modified look-locker inversion recovery (MOLLI) method with a 5b(3b)3b/4b(1b)3b(1b)2b scan scheme. The acquisition planes of the cine imaging included LV short-axis as well as two-chamber and four-chamber long-axis views. The scanning parameters included repetition time: 3.2 ms; echo time: 1.43 ms; flip angle: 44°; temporal resolution: 40 ms; field of view: 320 mm × 400 mm; acquisition matrix: 126 × 224; short-axis slice thickness: 8 mm with 8 to 10 slices acquired for full LV coverage. Myocardial T1 mappings were performed before and 15 min after gadolinium-diethylenetriaminepentaacetic acid (GD-DTPA) injection. The image planes included two-chamber view, four-chamber long-axis view and three short-axis views of LV at base, middle and apex. The scanning parameters included repetition time: 277 ms;echo time1 ms; flip angle 35°; field of view: 320 mm × 400 mm; acquisition matrix: 125 × 256. The contrast agent dose was 0.2 mmol/kg at an injection rate of 2.5 ml/s.

### Image analysis

All strain parameters and conventional cardiac function parameters were obtained using the commercial post-processing software cvi42 (Circle Cardiovascular Imaging, Calgary, Alberta, Canada, version 5.9.3). Cine image series acquired in short-axis and four-chamber long-axis orientations were imported into the 3D module of the software. Cine image series acquired in short-axis, two-chamber long-axis and four-chamber long-axis orientations were imported into the Tissue Tracking module of the software. Endocardium and epicardium tissue contours were drawn semi-automatically, and left and right ventricle boundary points were marked manually. Based upon the demarcations of LV, myocardial strain parameters were calculated via the cvi42 software package including: 2D and 3D RS, CS, and LS. Conventional cardiac function parameters were also calculated including LVEDVI, LVESVI, LVSVI, and LVMI. T1 values of the heart muscle and blood pool before and after enhancement were obtained from the inline T1 map generated by the MOLLI sequence. According to the LV 17-segment analysis method developed by the American Heart Association, the initial T1 time and post-enhancement T1 time of each segment were measured, and the mean T1 values before and after enhancement of segments 1–6 (basal segment), segments 7–12 (middle segment), and segments 13–16 (apical segment) were calculated. The ECV values of the LV basal segment, central segment and apex segment and the overall ECV values were calculated according to the following formula: ECV = (1-hematocrit) × [(1/T1_MyoPost_- 1/T1_MyoPre_)/(1/T1_BloodPost_-1/T1_BloodPre_)]. T1_MyoPre,_ T1_MyoPost_, T1_BloodPre_, T1_BloodPost_ were the T1 measurements performed in myocardium and blood before and after contrast enhancement, respectively. Hematocrit was obtained by routine hematological examination of the patient. EDWT was obtained by measuring the wall thickness of left ventricular segments 1–16 at end diastole (mean value recorded for each patient).

### Statistical analysis

Statistical analysis was performed using the SPSS version 20 (IBM Corp, Armork, New York, USA). All continuous data were expressed as mean ± standard, and one-way analysis of variance (ANOVA) was used for multiple comparisons. Data that did not conform to a normal distribution were expressed as median and inter-quartile range with two-sided non-parametric test used for multiple comparisons. The CMR data acquired from patients before and after drug treatment were compared using a paired-sample t-test or non-parametric test of related samples if data did not conform to a normal distribution. Pearson correlation or Spearman correlation coefficients were calculated to evaluate the relationship between different parameters (Spearman for cases wherein data did not conform to normal distribution). *P* < 0.05 was considered statistically significant.

## Results

### Patient characteristics and LV cardiac function

In this study, 62 HHD patients were enrolled and divided into 3 groups according to their EF. Among them, 22 patients in the group had an EF less than 30%, 18 patients had an EF greater than or equal to 30% and less than 50%, and 22 patients had an EF greater than 50%. Among these patients, 21 patients underwent CMR re-examination after taking antihypertensive drugs for more than 6 months. The antihypertensive drugs were either ACEI or ARB drugs. For these patients, after antihypertensive drug treatment, blood pressure was controlled to within 130–110/90–70 for twice consecutive observations. Table 1 lists the basic characteristics and main cardiac function parameters measured for HHD groups and control group. There were no statistically significant differences in age among the four groups. There was no significant difference between the EF value measured in HHD groups and the control group. LVEDVI, LVESVI, LVMI and EDWT of HHD patients increased with the decrease of EF, whereas, LVSVI didn’t show such trend. LVSVI of the HHD patients in the group with 30% < EF < 50% and the group with EF≧50% was increased compared to the control group, but LVSVI of the group with EF < 30% showed no significant difference from the control group.

**Table 1:** Patient Characteristics and LV function for HHD and healthy control groups

	HHD(*n* = 62)			Controls (*n* = 26)	*P* value
	EF < 30% (*n* = 22)	30%≦EF < 50% (*n* = 18)	EF≧50 (*n* = 22)		
Age (years)	43.7 ± 13.6	46.3 ± 14.8	49.3 ± 10.3	44.6 ± 13.7	0.36
Gender	20 M, 2F	16 M, 2F	19 M, 3F	22 M, 4F	0.28
BMI	30.4 ± 5.9	25.5 ± 3.5	25.7 (4.5)	24.6 ± 3.7	< 0.05^a d^
EF (%)	22.0 (6.6)	35.8 (14.6)	68.3 (9.3)	61.0 (7.3)	< 0.01^a b e f^
LVEDVI (ml/m^2^)	149.9 (50.1)	110.4 (43.2)	68.3 (24.7)	59.7 (23.3)	< 0.01^a b e f^
LVESVI (ml/m^2^)	122.1 (37.9)	70.2 (19.1)	21.3 (16.0)	23.4 (10.0)	0.000^a b e f^
LVSVI (ml/m^2^)	32.0 (15.3)	45.2 (22.6)	45.5 (13.3)	35.6 (14.1)	< 0.05^c d e^
LVMI (g/m^2^)	128.3 (40.4)	115.35 (39.1)	89.5 (35.6)	44.7 (13.9)	< 0.01^a b c e^
EDWT (cm)	1.2 ± 0.2	1.2 ± 0.2	1.1 ± 0.2	0.8 ± 0.2	< 0.05 ^a b c^

Abbreviations: ^a^ EF < 30% compared with Controls; ^b^ 30%≦EF < 50% compared with Controls; ^c^ EF≧50 compared with Controls; ^d^ EF < 30% compared with 30%≦EF < 50%; ^e^ EF < 30% compared with EF≧50; ^f^ 30%≦EF < 50% compared with EF≧50

### ECV

**Fig. 1:**
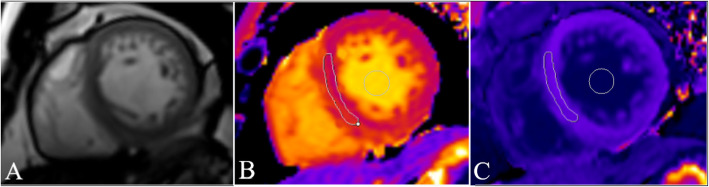
Representative example of T1 mapping for ECV measurement. Short-axis cine image (**a**), baseline pre-contrast T1 map (**b** with mean T1 relaxation time in myocardium ROI = 1272 ms and blood pool ROI = 1597 ms) and post-contrast T1 map (**c** with mean T1 relaxation time in myocardium ROI = 532msand blood pool ROI = 332 ms) and ECV = 0.22

Representative CMR images and T1 maps used for ECV calculations are shown in Fig. 1. Comparisons been the mean value and local ECV for each group are shown in Table 2. The mean ECV value of myocardium, and the ECV value of basal and middle myocardium of HHD groups with different EF were all higher than that of the normal control group (*P* < 0.05), but there was no significant difference among HHD groups. There was no significant difference of the ECV value in apical myocardium among the groups.

**Table 2:** ECV for HHD and healthy control groups

	HHD(*n* = 62)			Controls (*n* = 26)	*P* value
	EF < 30% (*n* = 22)	30%≦EF < 50% (*n* = 18)	EF≧50 (*n* = 22)		
Mean	0.28 ± 0.03	0.27 ± 0.03	0.27 ± 0.03	0.24 ± 0.02	< 0.05^a b c^
Base	0.30 (0.06)	0.29 (0.07)	0.28 (0.07)	0.23 (0.03)	< 0.01^a b c^
Middle	0.29 ± 0.05	0.29 ± 0.05	0.28 ± 0.03	0.24 ± 0.02	< 0.05^a b c^
Apex	0.25 (0.00)	0.25 (0.01)	0.25 (0.01)	0.25 (0.04)	0.992

Abbreviations: ^a^ EF < 30% compared with Controls; ^b^ 30%≦EF < 50% compared with Controls; ^c^ EF≧50 compared with Controls; ^d^ EF < 30% compared with 30%≦EF < 50%; ^e^ EF < 30% compared with EF≧50; ^f^ 30%≦EF < 50% compared with EF≧50

### Myocardial strain parameters

Comparisons between global and local myocardial strain values in the different groups are shown in Table 3. For HHD patients, with a decrease in EF the absolute value of strain decreased both globally and locally. There were statistically significant differences in GRS among the four groups (*P* < 0.01). After the exception of the GRS parameter, there was no significant difference in HHD in the EF≧50% group and the control group (*P* < 0.01). There were significant differences of the global and local CS and LS between the EF < 50% group and the EF≧50% group.

**Table 3:** Global and regional myocardial strain parameters for HHD and healthy control groups

	HHD(*n* = 62)			Controls (*n* = 26)	*P* value
	EF < 30% (*n* = 22)	30%≦EF < 50% (*n* = 18)	EF≧50 (*n* = 22)		
RS					
Global	8.2 ± 2.9	13.8 ± 4.3	29.1 ± 6.0	35.6 ± 7.5	< 0.01^a b c e d f^
Base	9.6 (7.3)	16.9 (10.4)(14.5817.39)	30.4 (17.0)	43.2 (17.0)	< 0.05^a b e f^
Middle	6.6 (4.0)	10.3 (5.2)	25.4 (8.9)	31.9 (14.2)	< 0.01^a b e f^
Apex	8.6 ± 5.9	13.7 ± 3.9	31.3 ± 11.1	36.2 ± 10.9	< 0.01^a b e f^
CS					
Global	−7.1 (3.2)	−10.1 (3.2)	−19.6 (4.8)	−20.5 (4.1)	< 0.01^a b e f^
Base	−6.5 (2.5)	−10.1 (3.2)	−17.5 (5.8)	−18.9 (3.8)	< 0.01^a b e f^
Middle	−6.2 (3.0)	−10.8 (3.3)	−20.2 (3.9)	−20.7 (4.2)	< 0.01^a b e f^
Apex	−9.2 (4.9)	−12.6 (5.1)	−22.5 (5.2)(24.74)	−22.8 (5.3)(23.29)	< 0.01^a b e f^
LS					
Global	−4.0 (2.9)	−6.5 (2.4)	−12.1 (3.8)	−14.9 (3.6)	< 0.05 ^a b e f^
Base	−2.7 (7.3)	−4.7 (4.5)	−10.4 (6.2)	−12.6 (4.8)	< 0.05 ^a b e f^
Middle	−3.7 (3.2)	−6.5 (3.6)	−12.1 (6.0)	−14.2 (4.7)	< 0.05 ^a b e f^
Apex	−5.4 (2.9)	−10.0 (3.0)	−14.9 (3.4)(35.55)	−17.9 (4.1)	< 0.05 ^a b e f^

Abbreviations: ^a^ EF < 30% compared with Controls; ^b^ 30%≦EF < 50% compared with Controls; ^c^ EF≧50 compared with Controls; ^d^ EF < 30% compared with 30%≦EF < 50%; ^e^ EF < 30% compared with EF≧50; ^f^ 30%≦EF < 50% compared with EF≧50

### Relationships between LV function parameters, ECV, and myocardial strain

**Fig. 2:**
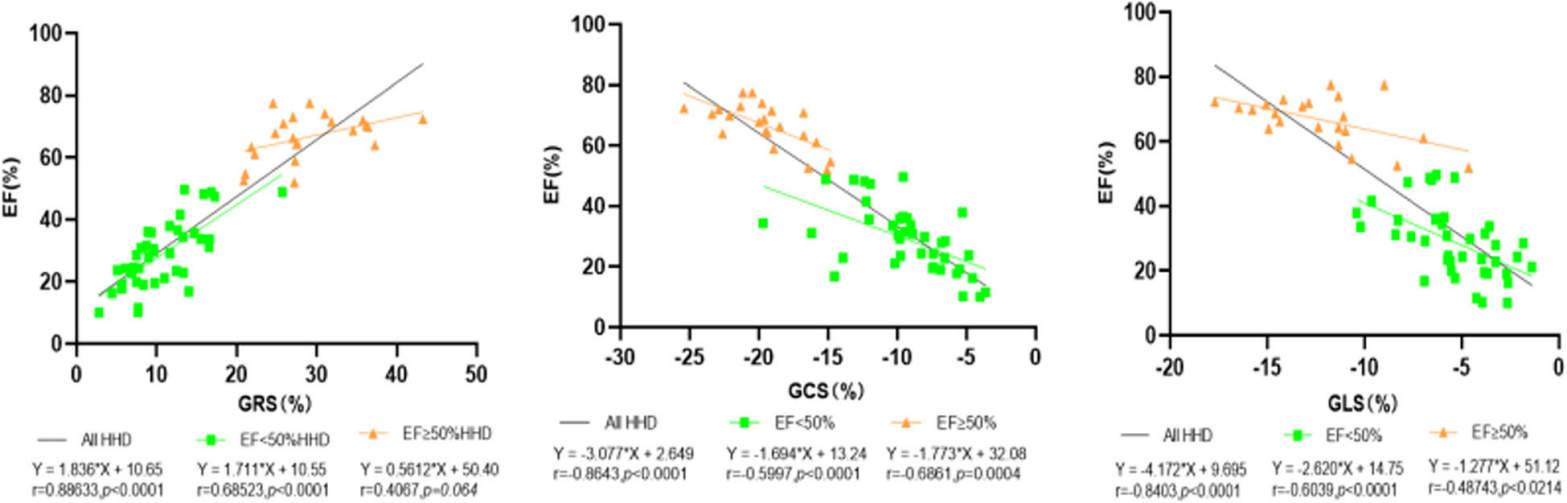
Relationship between EF and myocardial strain measurements. There was strong correlation between EF and GRS, GCS, and GLS strain measurements. When separately considering only those patients with EF less than 50%, the magnitude of slope between EF and GLS, GCS, and GLS parameters was greater than observed for patients with EF≧50%

When comparing all HHD patients, EF demonstrated a significant correlation with GRS, GCS, and GLS strain parameters (Fig. 2). The coefficient of association between.

**Fig. 3:**
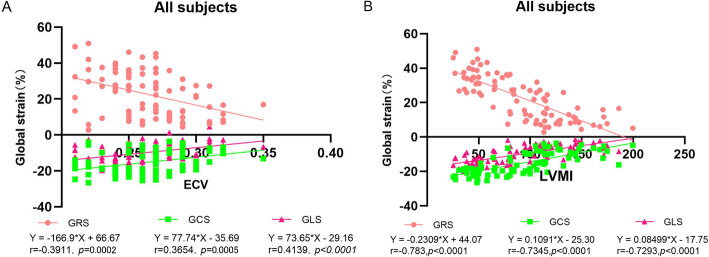
The relationship between myocardial strain and ECV as well as that between myocardial strain and LVMI. A relatively weak correlation was observed between ECV and global strain (A). A strong correlation was observed between LVMI and global strain (B)

EF and GRS is the biggest in patient groups with EF < 50%; however, GLS has the greatest effect on EF for the largest slope. In patient groups with EF ≧ 50%, GCS has a larger slope and a larger correlation coefficient than that of GRS and GLS. Across all the subjects, global strain parameters demonstrated a relatively weak correlation to ECV (Fig. 3a). A strong correlation was observed between LVMI and global strain parameters (Fig. 3b).

### Comparison between LV function, ECV and myocardial strain before and after treatment

**Fig. 4:**
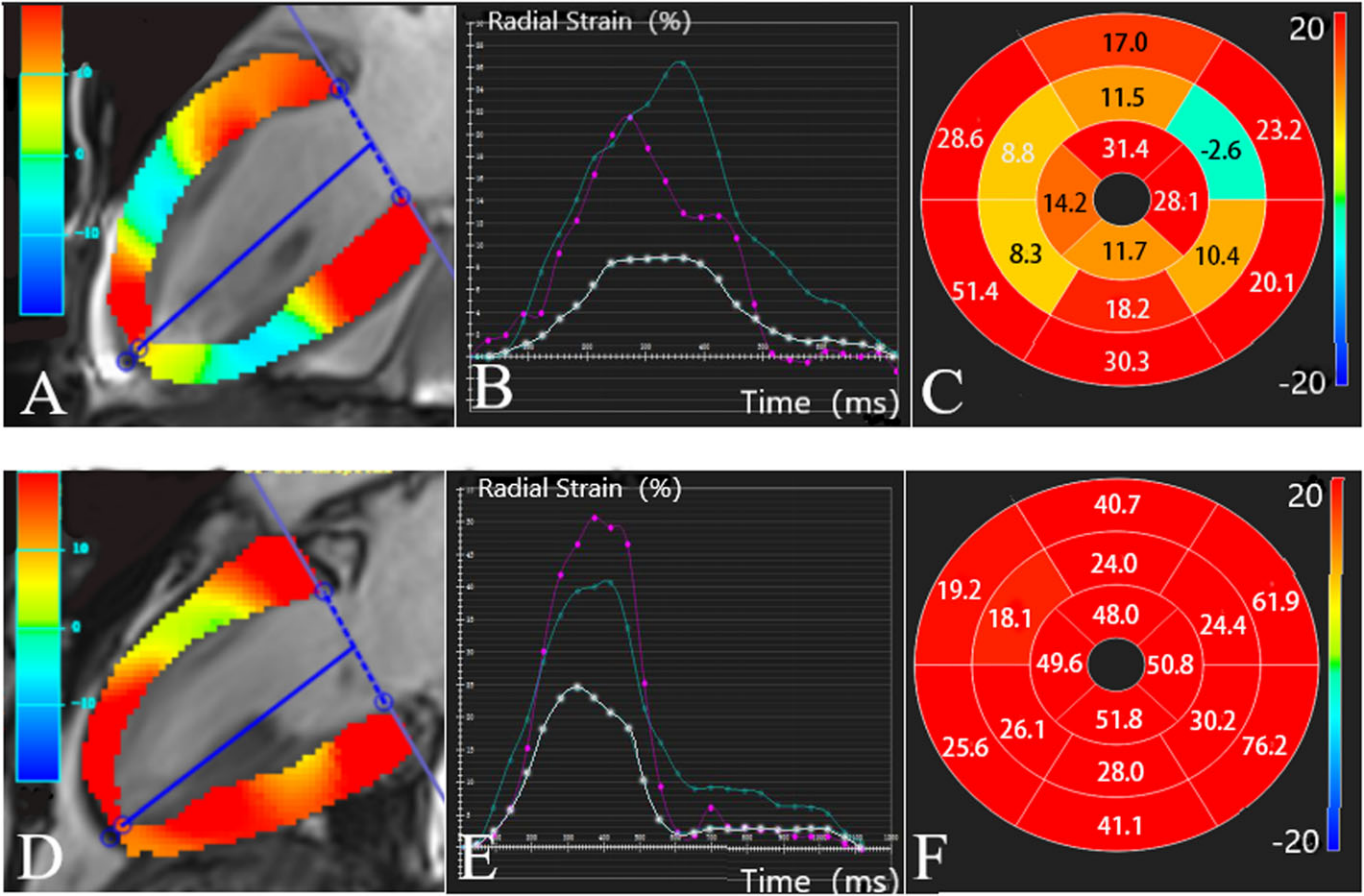
RS comparison before and after treatment of a HHD patient. Long-axis left-ventricle views in HHD patient before (**a**) and after (**d**) 6-month of treatment with color-map overlay of RS measurements at end systole. The corresponding time course of RS measurements across the cardiac cycle for basal (green line), middle (white line), and apical (purple line) segments are shown in panels **b** and **e**, respectively. RS measurements before (**c**) and after (**f**) treatment for each segment of the 16-segment model are shown

Overall, after 6 months of antihypertensive drug treatment, patients had higher EF (*P* = 0.02), lower LVEDV (*P* = 0.03), EDWT (*P* = 0.03), and mean ECV (*P* < 0.001). The RS of global and middle segment and the absolute value of the basal and global CS were all increased (*P* < 0.05). The differences among LVEDVI, LVSVI, LVMI, and LS were not statistically significant (Table 4). As shown in Fig. 4, the RS of a patient with HHD was improved after 6 months treatment. The strain of apical, middle, and basal segments tended to be synchronized and recovered after treatment (Fig. 4e).

**Table 4:** Comparison between LV function, ECV and myocardial strain before and after 6-months anti-hypertension drug therapy

	Before treatment	After treatment	*P* value
EF	44.6 ± 22.3	52.4 ± 18.9	0.02
LVEDVI (ml/m2)	96.9 (67.7)	74.8 (40.1)	0.06
LVSVI (ml/m2)	40.0 ± 16.4	40.1 ± 13.8	0.85
LVMI(g/m2)	106.5 (31.2)	97.6 (36.2)	0.18
EDWT (cm)	1.2 (0.2)	1.1 (0.3)	0.03
ECV			
Mean	0.27 (0.04)	0.25 (0.04)	0.000
Base	0.29 ± 0.04	0.25 ± 0.04	0.000
Middle	0.25 (0.01)	0.24 (0.01)	0.001
Apex	0.25 (0.01)	0.24 (0.01)	0.71
RS			
Global	16.8 ± 8.9	22.7 ± 9.5	0.01
Base	20.7 ± 11.1	29.5 ± 16.7	0.06
Middle	11.0 (17.0)	20.2 (14.3)	0.027
Apex	13.6 (17.9)	20.4 (22.3)	0.64
CS			
Global	−13.4 ± 5.6	−16.0 ± 4.6	0.03
Base	−9.9 (10.5)	−10.9 (7.8)	0.02
Middle	−12.0 (11.9)	−17.4 (8.3)	0.16
Apex	−16.2 ± 6.5	−19.6 ± 5.2	0.05
LS			
Global	−6.9 (6.0)	−9.0 (5.4)	1.00
Base	−5.4 (5.0)	−5.2 (13.1)	0.13
Middle	−6.4 (6.4)	−8.7 (5.8)	0.50
Apex	−10.7 (8.0)	−12.0 (6.3)	0.12

## Discussion

In this study, we made several important findings. Firstly, after 6 months of antihypertensive drug therapy, there was an improvement in cardiac function, myocardial fibrosis, and LVH in HHD patients with well-controlled blood pressure. Secondly, GRS may be more sensitive to assess the systolic function of HHD compared with other parameters. Thirdly, compared with myocardial fibrosis, myocardial hypertrophy has better correlation with myocardial strain. In addition, there is a local difference in the elevation of ECV in HHD patients. Compared with the apical segment of LV, the middle segment and the basal segment may be more likely to be involved in the HHD.

### ECV

There was no significant difference in ECV among the groups with different EF (Table 2), but we found that there was a local difference in ECV. ECV values in the basal and middle segments were higher than those in the apical segment; however, there was no such difference in the normal control group, suggesting that HHD fibrosis may be more easily involved in the basal and middle segments. The ECV values are overlapped among the groups. Therefore, it is difficult to distinguish HHD patients with preserved EF from the normal control group only by the ECV value.

### Myocardial strain

The absolute value of the global and local strain decreased with the decrease of EF in HHD patients. The myocardial strain value was also reduced in HHD patients with preserved EF value, suggesting that the strain value is more sensitive in reflecting the LV systolic function compared to EF value. Among the three global strains, GRS has the greatest value change in HHD patients with EF≧50%. That is to say, GRS may be more sensitive to evaluate systolic function for these patients, consistent with literature report [14]. By combining mathematical and echocardiographic methods, Stokke Thomas M et al. found that strain measurement seems to be a better method for quantifying systolic function, and that longitudinal shortening may be a more sensitive marker of systolic dysfunction [15], which may be related to the diseases we study and the different examination methods. We found that the GCS contributed more than GRS and GLS to EF in the HHD patients with preserved EF, though it didn’t change much. Studies have shown that significant reduction of LS can be compensated by increasing CS or wall thickness or the decrease of diameter [15]. In addition, although the GRS value decreased, the slope of the univariate linear regression between GRS and EF is small, so that the effect on EF is weak. This may be the reason why the EF can keep normal.

### Correlation

We found that LVMI was positively correlated with the GCS and GLS but negatively correlated with GRS. Compared to the correlation between ECV and LVMI, the correlation between myocardial strain and LVMI was stronger. It is consistent with the literature reports that LVH has a greater effect on overall LS than diffuse fibrosis [16]. Studies have found that ECV in hypertensive cardiomyopathy is positively correlated with LV mass and CS [17]. An animal study showed that diffuse subendocardial fibrosis is also correlated with decreased longitudinal contractile force and decreased peripheral strain in hypertensive rats [18]. Furthermore, although the correlation coefficient was low, we still observed a correlation between ECV and strain in all subjects, suggesting that diffuse myocardial fibrosis causes the reduction of the myocardial strain. Previous studies have also reported that an increase in ECV reflects potential changes, such as fibrosis, collagen swelling, and increased collagen cross-linking, leading to changes in myocardial function [19].

### Impact on LV function parameters, ECV, myocardial strain after treatment

Studies have shown that LVH regression by inhibiting the neuroendocrine pathways of rennin angiotensin aldosterone system (RASS) and sympathetic nervous system (SNS) is greater than LVH regression by lowering blood pressure alone [20]. ACEI and ARB antihypertensive drugs can inhibit the RASS neuroendocrine pathway and improve LV remodeling [8, 20]. After six-month treatment, the mean wall thickness at the end of the diastolic period was significantly improved, but the changes in LVMI and LVEDVI were not significant, suggesting that short-term treatment improved LVMI and LVEDVI weakly. After six-month treatment, the EF value and the absolute value of myocardial strain increases, indicating that myocardial systolic function is improved. Besides, there were obvious improvements of GRS and GCS after treatment, but there was no improvement of GLS. This suggests that GRS and GCS may be more sensitive to the treatment. Although the improvement of LS impairment in HHD patients is difficult, perhaps it is more important for prognostic evaluation. A study on HHD echocardiography showed that strain (especially GLS) was associated with the major adverse cardiac events (MACE), and the MACE was also associated with the deterioration of LV longitudinal function during mid-term follow-up [21]. After treatment, there was a definite but limited decrease in the ECV value of HHD patients, suggesting that the myocardial fibrosis may be slightly improved after treatment.

### Limitations

The current study has some limitations. First of all, the sample size is small, so the sample may not accurately reflect the overall situation. Secondly, there is no grouping based on the choice of treatment drugs, so different types of antihypertensive drugs may have different therapeutic effects, and CMR parameters may also change differently. Thirdly, patients were not followed up for cardiac adverse events after treatment, and the combination of changes in related parameters with prognosis may be more meaningful for guiding clinical treatment. In addition, molecular, biomechanical and structural changes in HHD after treatment may take a longer time. Hence, the changes of the study metrics may be more significant had the study duration been prolonged.

## Conclusions

ECV and myocardial strain parameters for myocardial abnormalities are more sensitive. ECV, GRS and GCS are more sensitivity to treatment than other parameters, however the improvement of GLS in patients with HHD is weak. ECV and myocardial strain parameters can be used as CMR marker for long-term monitoring of the curative effect of patients with HHD.

## Data Availability

The datasets used and analysed during the current study are available from the corresponding author on reasonable request.

## Abbreviations

CMR: Cardiovascular magnetic resonance imaging
HHD: Hypertensive heart disease
LV: Left ventricular
ECV: Extracellular volume
EF: Ejection fraction
LVH: Left ventricular hypertrophy
LVMI: Left ventricular mass index
LVEDVI: Left ventricular end-diastolic volume index
LVESV: Left ventricular end-systolic volume index
LVSVI: Left ventricular stroke volume index
EDWT: End diastolic wall thickness
RS: Radial strain
CS: Circumferential strain
LS: Longitudinal strain
ACEI: Angiotensin converting enzyme inhibitor
ARB: Angiotensin receptor blocker
